# Root-analogue implants compared to forced orthodontic extrusion: a retrospective analysis of clinical, radiological and esthetic outcomes after restoration

**DOI:** 10.1007/s00784-023-05198-6

**Published:** 2023-08-15

**Authors:** Mats Wernfried Heinrich Böse, Florian Beuer, Michael Naumann, Benedikt Christopher Spies, Stefan Neumeyer, Detlef Hildebrand, Maria Bruhnke

**Affiliations:** 1 Department of Prosthodontics, Geriatric Dentistry and Craniomandibular Disorders, Charité – Universitätsmedizin Berlin, corporate member of Freie Universität Berlin, Humboldt-Universität zu Berlin, and Berlin Institute of Health, Aßmannshauser Str. 4-6, 14197 Berlin, Germany; 2 Medical Center – University of Freiburg, Center for Dental Medicine, Department of Prosthetic Dentistry, Faculty of Medicine, University of Freiburg, Hugstetter Straße 55, 79106 Freiburg, Germany; 3 Private Dental Practice, Gemeinschaftspraxis Dr. Stefan Neumeyer & Partner, Leminger Str. 10, 93458 Eschlkam, Germany; 4 Private Dental Practice, Dr. Detlef Hildebrand, Westhafenstraße 1, 13353 Berlin, Germany

**Keywords:** Prosthodontics, Endodontically treated tooth, Forced eruption, Orthodontic extrusion, Dental implants, Root-analogue implants

## Abstract

**Objectives:**

To assess clinical, radiological and esthetic outcomes of restorations supported by root-analogue implants (RAIs) or roots of severely damaged teeth after forced orthodontic extrusion (FOE).

**Materials and methods:**

Clinical data regarding milled one-piece (titanium/zirconia roots and zirconia abutments) RAIs (REPLICATE™ System) and FOE were recorded and retrospectively evaluated for 40 patients by two investigators. Strict inclusion and exclusion criteria were applied. Functional and esthetic outcomes were assessed for *n* = 20 pre-molars and *n* = 20 anterior teeth via comparison of radiographic and digital images applying the novel Functional Implant Prosthodontic Score (FIPS). Krippendorff’s alpha coefficient was calculated to assess inter-rater reliability. Mann–Whitney-U-Test was used to compare the assessed parameters. Level of significance was set to *p* < 0.05.

**Results:**

After a mean observation period of 18.4 ± 5.7 months for restorations supported by RAIs and 43.9 ± 16.4 months for restorations after FOE, mean FIPS scores were 9.2/8.8 ± 1.1/1.2 (RAIs) and 7.4/7.7 ± 1.3/1.5 (FOE), respectively. Krippendorff’s alpha coefficients did not reveal unacceptable inter-rater reliabilities regarding the investigators and applicability of FIPS. Significant differences were documented when comparing restorations after FOE or supported by RAIs regarding bone loss (*p* < 0.01), presence of papillae (*p* < 0.05) and quality and quantity of mucosa (*p* < 0.02) in favor of FOE.

**Conclusions:**

Within the main limitations of sample size and the retrospective study design, both concepts seem to provide clinically acceptable results.

**Clinical relevance:**

Bone- and tissue-preserving characteristics regarding the concept of FOE are promising. It could be applicable for socket preservation and subsequent conventional implant placements in an adapted workflow.

## Introduction

In case of extensively destroyed teeth, dentists must regularly decide between tooth preservation and extraction [1, 2]. Regarding this decision, future prosthodontic treatment options to restore function and esthetics should be considered. For the restoration of single-tooth gaps, conventional treatment options such as fixed dental prostheses (FDPs) [3–5], resin-bonded fixed dental prostheses (RBFDPs) [6–8] and implant-retained restorations [3, 9–13] have been established in daily dental routine. Usually, at this clinical state, teeth have already been removed or are expected to be extracted. However, it is well known that tooth extraction is accompanied by remodeling processes of the surrounding hard and soft tissues whereby volume is usually lost [14]. This can lead to restorative limitations in general and become a challenge, especially in the esthetic zone. To counteract these resorption processes after extraction, immediate implant placement continues to be controversially discussed [15–21]. Thereby, root-analogue implants (RAIs) represent a highly individual procedure of immediate dental implant installations.

The concept of RAIs was first scientifically described in 1969 with the Dental Polymer Implant Concept by Hodosh et al. [22]. Thereby, RAIs were fabricated from polymethyl methacrylate (PMMA) after extraction with a transfer technique using the removed roots and plaster to copy the anatomical shape. However, connective tissue healing of PMMA RAIs led to the discontinuation of the concept [23]. At the beginning of the 90 s, the idea was revisited and experiments in beagle dogs with roots copied by machine and made of titanium were conducted [24]. After two, twelve and 36 months, the evaluation of clinical, radiographic and histological parameters showed successful osseointegration of 88% of 32 duplicates. Consequently, in the late 90 s, a research group led by Strub and Kohal et al. introduced the "Re-Implant System" (Re-Implant GmbH, Hagen, Germany) [25]. The extracted roots were fabricated from titanium using a milling process, but clinical follow-up in 2002 presented an unsatisfactory survival rate of 48%. By the end of the 2000s, RAIs could be fabricated using modern computer-aided design/computer-aided manufacturing (CAD/CAM) technologies. Again, extracted teeth were used as basis for fabrication and in a two-year clinical study, a survival rate of 92% was documented for the so-called "BioImplant Concept" with RAIs made from zirconia [26]. Finally, the introduction of cone beam computed tomography (CBCT) into dentistry allowed for a prefabrication of RAIs, making immediate implant installation possible without a time delay between extraction and insertion [27]. Recently published data revealed stable peri-implant soft tissue conditions and satisfying esthetic outcomes regarding RAIs with a survival rate of 94.4% after a short-time observation period of 18.9 ± 2.4 months [28]. Nevertheless, clinical studies on RAIs are scarce, especially compared to those for screw-shaped implants. Therefore, more data including different manufacturing techniques and biomaterials are regularly requested in current literature [29–36].

On the other hand, to eliminate the concerns regarding resorption processes, the possibility of tooth/root preservation and restoration should be discussed as a viable treatment option. Thereby, especially the size of defects and subgingival restoration margins can be problematic by affecting the patient’s periodontal health [37] as they might violate biologic width [38]. Additionally, scientific literature demands a circumferential ferrule design preparation for long-term success of restorations [39, 40]. To re-establish biologic width and to facilitate a circumferential ferrule design preparation, pre-prosthetic treatment protocols such as surgical crown lengthening [41] or forced orthodontic extrusion (FOE) procedures [42, 43] have been suggested in the literature. Surgical crown lengthening is an operative procedure associated with an osseous reduction of the alveolar bone and an inevitable lengthening of the clinical crown [44]. This might lead to esthetic deformities, which poses an esthetic problem in the anterior zone. In contrast, FOE is a valid treatment alternative [44] maintaining soft and hard tissues. Therefore, the procedure is regarded maximally tissue preserving and minimally invasive [45]. Extrusion is a defined orthodontic movement in occlusal direction. It enables the re-establishment of biologic width and exposes sound tooth structure to facilitate placement of dental restorations [46]. Orthodontic extrusion is indicated for teeth with horizontal, shear or cuspal fractures, carious destruction, resorption and iatrogenic perforations [46]. Although the treatment procedure of FOE was described as early as in 1973 [42] scientific evidence is currently limited to few studies [43, 47, 48]. Numerous articles have been published demonstrating different approaches of orthodontic extrusion, as by fixed orthodontic arch wires and elastics [49], removable orthodontic appliances [50], existing removable partial dentures [51] as well as complete dentures [52]. Scientific evidence on the long-term prognosis for teeth after FOE is scarce. However, there are two clinical studies reporting on favorable survival rates after a short time of observation and concluding orthodontic relapse as the major complication of this technique [48, 53].

The aim of this retrospective investigation was to evaluate and compare clinical, radiological and esthetic outcomes of prosthodontic rehabilitations supported by RAIs or natural roots after FOE. The working hypothesis was, that both treatment concepts show comparable results.

## Materials and methods

### Study design and ethical approval

For the present retrospective investigation, available clinical data regarding restorations supported by RAIs or natural roots after FOE were retrospectively evaluated and compared. Ethical approval was given by the local Ethical Committee of Charité – Universitätsmedizin Berlin, Germany (application numbers: EA4/140/18 and EA2/301/20). All RAI-treatments were performed by the author D.H. and respective follow-up examinations by the author M.W.H.B. All FOE-treatments and follow-up examinations were performed by the author M.B. To reduce subjective bias, analyzed outcomes were evaluated independently by both first and last specified authors of this article. All patients have received and signed a written informed consent form and patient information. It was conducted considering the STROBE statement for observational studies (https://www.strobe-statement.org) where applicable.

For treatments with a RAI, the following criteria regarding the patients had to be fulfilled: 1) Non-smokers; 2) No medication affecting the bone metabolism; 3) Non-inflammatory surrounding soft and hard tissues and 4) Bone compartments surrounding the relevant tooth had to be intact. For FOE following inclusion criteria were defined: 1) Patients in need of a restoration of a deeply destroyed tooth with two adjacent teeth; 2) Probing depths ≤ 2 mm and defects violating the biologic width and/or a missing ferrule design preparation; 3) Prospective crown-to-root ratio ≤ 1; 4) Tooth mobility ≤ 1 and prospective single-tooth restorations. Teeth with suspicion of hypercementosis/ankylosis, molars and teeth with vertical root fractures were excluded. Treatments were only performed in patients whose compliance regarding the necessary monitoring was expected.

Due to the retrospective comparative study design, the cases to be evaluated had to be selected based on the available data. Therefore, *n* = 10 pre-molars restored with single crowns supported by RAIs, *n* = 10 severely damaged roots of pre-molars restored with single crowns after FOE, *n* = 10 anterior teeth (canine to canine) restored with single crowns supported by RAIs and *n* = 10 severely damaged roots of anterior teeth restored with single crowns after FOE were selected. Thus, 40 cases could be included. Available data were anonymized, retrospectively evaluated and statistically analyzed. Post-hoc power-analysis was performed with a free-to use software to calculate statistical power (G* Power 3.1.9.7, Heinrich-Heine-Universität Düsseldorf, Düsseldorf, Germany) with a sample size of *n* = 20 per group, *α* = 0.05 and an effect size of 1.17 resulting in a power of 0.97 (97%).

### Root-analogue implants (RAIs)

**Fig. 1:**
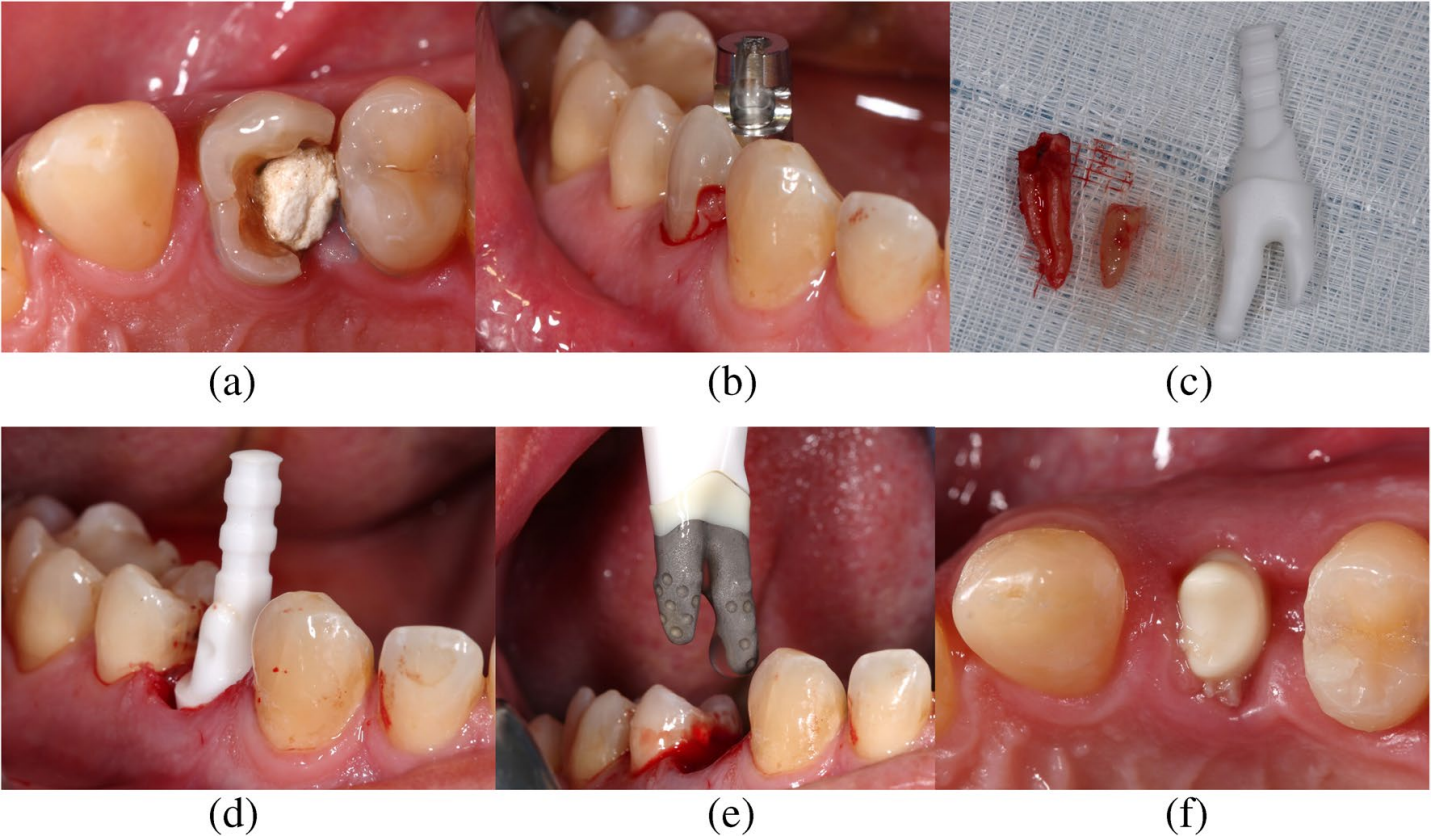
Clinical workflow regarding the extraction of a deeply destroyed tooth (FDI 24) and exemplary immediate implant placement of a RAI. **a** Initial clinical situation (FDI 24); **b** Application of the Benex Extraction-System; **c** Extracted roots and Try-In; **d** Testing the expected fit with Try-In; **e** Insertion of milled one-piece hybrid RAI and (**f**) Clinical situation after healing period. Abbreviations: (1) FDI: Fédération Dentaire Internationale; (2) RAIs: Root-analogue implants

For rehabilitations with RAIs, these had to be manufactured individually before surgeries. Impressions were taken with customized trays and a polyether material (Impregum, 3 M Deutschland GmbH, Neuss, Germany). These were cast with type IV plaster and digitized with a laboratory scanner where after the model data were available as Standard Triangle/Tessellation Language (STL) datasets. Bite records were taken with an injectable elastomeric A-silicone (Futar D, KettenbachGmbH&Co. KG, Eschenburg, Germany) and DICOM data were exported from an x-ray device (PAX i-3D, VATECH, Hwaseong-si, Gyeonggi-do, South Korea). A highly trained specialist of the manufacturer superimposed the DICOM and STL data and designed the Temporary Protective Covers (TPCs), implant and abutment portions of the RAIs (Fig. 1e and f) and Try-Ins (exact copies of the RAIs made from sterilizable resin, Fig. 1c and d). From resulting Computer Aided Design (CAD) data, TPCs and RAI components were milled in a Computer Aided Manufacturing (CAM) process. All ceramic parts (abutment portion, root portion in case of all-ceramic RAIs and TPCs) were made of yttria-stabilized tetragonal polycrystalline zirconia (Y-TZP) and sintered. With hybrid RAIs, root portion was manufactured from pure titanium (medical grade IV) and bonded to the aforementioned abutment portions made from Y-TZP in a special oven process using a biocompatible glass material without any voids. These connections were checked using x-rays within quality control. Afterwards, root portions of both (all-ceramic and hybrid RAIs) were modified with macro- and micro retentions (medical grade corundum and etched). The Benex Extraction-System (Benex Extraction-System, Helmut Zepf Medizintechnik GmbH, Seitlingen-Oberflacht, Germany, Fig. 1b) was used to remove teeth minimally invasive in vertical direction. If this was not successful, teeth were carefully removed using conventional extraction techniques. Intact surrounding bone compartments were obligatory. The expected fit of RAIs was tested by using the respective Try-Ins (Fig. 1d). Before implant insertion, implant surfaces and alveoli were wetted with Plasma Rich Growth Factors (PRGFs; BTI Biotechnology Institute, San Antonio, Spain). RAIs were carefully placed (Fig. 1e) and present voids were filled with PRGFs as well. As load protection TPCs were semi-adhesively attached (RelyX Ultimate, 3 M Deutschland GmbH) to one or both adjacent teeth depending on the design. This provided a protective gap of approx. 0.6 mm between the abutment portion of RAIs and TPCs it selves. The workflow has already been described and illustrated in detail in a publication in 2020 [28] and is additionally shown in Fig. 1. Healing was usually checked 3–6 month after surgeries using clinical and radiological parameters. Prosthodontic treatments were performed in the office of the author D.H. and different referring dentists, whereby no constantly defined workflow was followed. In total, 16 hybrid and four all-ceramic RAIs with respective restorations were investigated.

### Forced orthodontic extrusion (FOE)

**Fig. 2:**
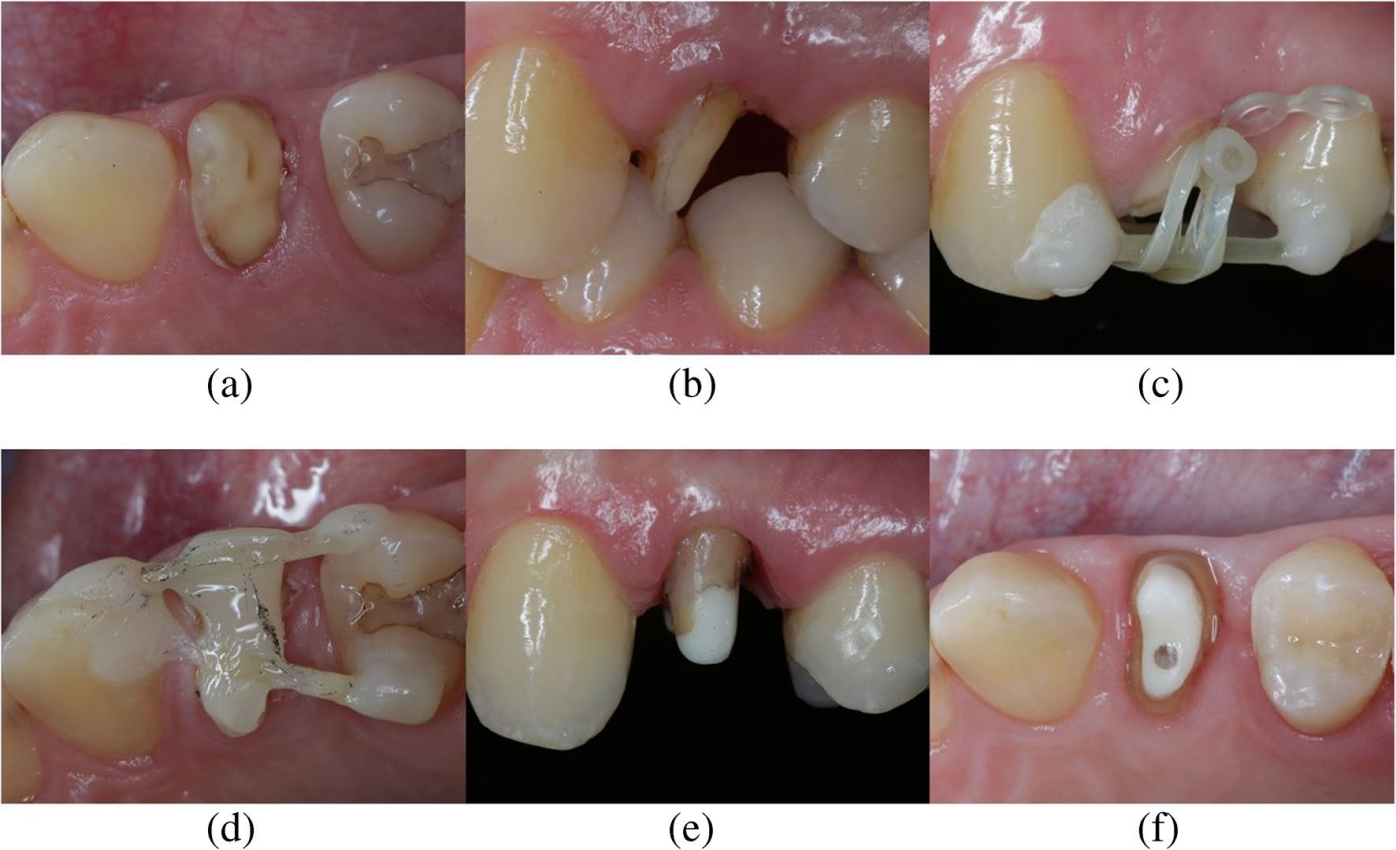
Clinical workflow regarding the restoration of a deeply destroyed tooth (FDI 24) after FOE. **a** Initial clinical situation (FDI 24); **b** Buccal cusp of the tooth is missing; **c** Orthodontic elastics initiate tooth movement in occlusal direction; **d** Tooth is splinted to adjacent teeth after successful extrusion for time of retention; Final preparation from buccal (**e**) and from occlusal (**f**) shows a circumferential ferrule design preparation. Abbreviations: (1) FDI: Fédération Dentaire Internationale; (2) FOE: Forced orthodontic extrusion

Prior to orthodontic extrusion, probing depths, tooth mobility, defect size and radiographic images were assessed to determine the amount of extrusion and the prospective crown-to-root ratio for each patient individually. Interocclusal available space for orthodontic extrusion was analyzed with the aid of gypsum models. After removal of insufficient restorations, a fiber-reinforced composite-based post (Extrusion pin, Komet Dental, Lemgo, Germany) was placed on the root surface of respective teeth with self-adhesive resin (RelyX Unicem 2 Automix, 3 M Deutschland GmbH, Fig. 2c) in central vestibulo-oral direction, at the widest root diameter.

A second post serving as anchorage was adhesively bonded to adjacent teeth with flowable composite resin (Tetric EvoFlow, Ivoclar Vivadent AG, Schaan, Liechtenstein, Fig. 2c). Elastics were placed in the orthodontic appliance to initiate forced orthodontic extrusion with forces > 0.5N (Fig. 2c). At the same appointment, supra-crestal fibrectomy, scaling and root planning procedures were performed [54]. In some clinical cases two bars had to be bonded to adjacent teeth due to minor occlusal space. In clinical situations requiring crown restorations on neighboring teeth, anchorage was realized with the aid of a provisional FDP and a reduction of the pontic area to allow for a sufficient amount of extrusion. Clinical details on the workflow have already been described and published [47]. An exemplary workflow is additionally shown in Fig. 2. Elastics were changed by the patients twice daily due to the loss of tension. Patients were available for control visits once a week. The mean amount of extrusion was 3.50 ± 0.87 mm and the mean time of extrusion 17.88 ± 10.98 days. After extrusion, teeth were bonded to adjacent teeth with composite resin (Tetric EvoFlow, Ivoclar Vivadent AG, Fig. 2d) for a retention period of at least 8 weeks (mean time of retention 130.60 ± 89.12 days) to prevent orthodontic relapse [46]. After revision of endodontic fillings, placements of glass-fiber posts (X-Post, DentsplySirona, Bensheim, Germany) were performed where indicated, teeth were built up with composite resin and restored with single crowns.

### Study parameters

Within the framework of a feasibility analysis regarding this investigation, existing data sets were reviewed for completeness by the authors M.B. and M.W.H.B. to be able to compare restorations supported by RAIs or natural roots after FOE. The novel Functional Implant Prosthodontic Score (FIPS) was chosen [55] for evaluation and slightly modified regarding rehabilitations of natural roots after FOE not representing implant-retained restorations (Table 1). It was selected as it is a straightforward, self-explaining, reliable, reproducible and quickly applicable score [55, 56].

**Table 1:** Definition of the Functional Implant Prosthodontic Score (FIPS) with slight adjustments regarding the evaluation of FOE restorations [55]

Variables	0	1	2
Interproximal Contacts and papillae	Major discrepancy (2 × incomplete)	Minor discrepancy (1 × complete)	No discrepancy (2 × complete)
Occlusion Static and dynamic	Major discrepancy (supra-contact)	Minor discrepancy (infra-occlusion)	No discrepancy
Design Contour and color	Major discrepancy (contour/color deficiencies)	Minor discrepancy (color deficiencies)	No discrepancy
Mucosa Quality and quantity	Non-keratinized Non-attached	Non-keratinized attached	Keratinized attached
Bone X-ray	Radiographic bone loss > 1.5 mm (RAIs) or > 10% (FOE)	Radiographic bone loss < 1.5 mm (RAIs) or < 10% (FOE)	No radiographic bone loss
Maximum score			**10**

Five variables evaluating interproximal, occlusion, design, mucosa and bone including corresponding sub-categories

Abbreviations: (1) RAIs: Root-analogue implants; (2) FOE: Forced orthodontic extrusion

FIPS is defined by five variables, allowing to evaluate the interproximal area, the occlusion, the design, the mucosa and the bone. After rating every mentioned parameter with 0, 1 or 2, a maximum score of 10 can be achieved by a single restoration (Table 1). In case of subcategories (i.e. “interproximal” with “contacts” and “papillae”) with different ratings, the lowest has to be selected. Therefore, for evaluation, following data had to be present: 1) Photographs for the visual assessment of papillae, the design/esthetics of the restorations, and the mucosa; 2) Documented information regarding the interproximal contacts (checked with dental floss); 3) Documented information regarding the static and dynamic occlusion (checked with shimstock foil, thickness: 8 μm); 4) Available x-rays for the evaluation of marginal bone loss.

**Fig. 3:**
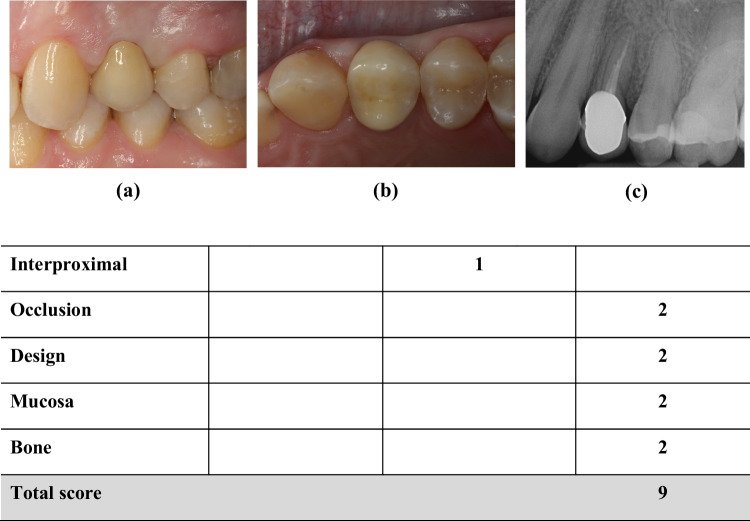
Study participant treated with a single-crown after FOE treatment in regio 24 (FDI). Illustration based on original publication of FIPS: **a** lateral and **b** occlusal views as well as (**c**) 2D radiographic image. Abbreviations: (1) FOE: Forced orthodontic extrusion; (2) FDI: Fédération Dentaire Internationale; (3) FIPS: Functional Implant Prosthodontic Score [55]

For evaluation of interproximal bone changes regarding restorations of severely damaged roots after FOE, radiographic images of teeth before intervention and at recall appointments were superimposed. Therefore, reference lines were drawn on apices and incisal edges of neighboring teeth. The root length of the extruded tooth was divided into ten equal segments. An interproximal bone change of one tenth in a section was defined as interproximal bone loss [57]. A stable interproximal bone level was rated with 2, a change in one tenth of a section was rated with 1 and an interproximal bone loss of more than one tenth of a section was rated with 0 (Fig. 3).

**Fig. 4:**
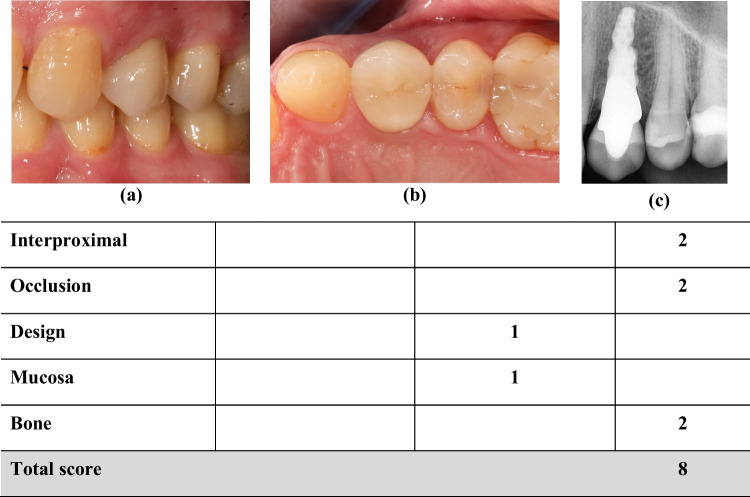
Study participant treated with a RAI-supported single-crown in regio 24 (FDI). Illustration based on original publication of FIPS: **a** lateral and (**b**) occlusal views as well as (**c**) 2D radiographic image. Abbreviations: (1) RAI: Root-analogue implant; (2) FDI: Fédération Dentaire Internationale; (3) FIPS: Functional Implant Prosthodontic Score [55]

Due to the availability of the implant lengths of RAIs, the evaluation of bone loss could be carried out in accordance with FIPS score after superimposition of pre- and post-loading x-rays using mathematical rule of three (Fig. 4). Total marginal bone loss without a view to the rough-smooth border between implant and abutment portions were rated. Due to the retrospective study design, no individual bite holders could be used for both investigated concepts.

### Statistical analysis

All statistical tests were performed by an independent examiner using “SciPy” (https://scipy.org/, last accessed 13th of March, 2022), a Python-based open source software environment mainly used for scientific analysis, visualizations and related activities. Krippendorff’s alpha coefficient was calculated to assess inter-rater reliability between the authors M.W.H.B. and M.B. Due to ordinal FIPS data, Mann–Whitney-U-Test was used to compare the assessed parameters regarding restorations of natural roots after FOE or supported by RAIs. Level of significance was set to *p* < 0.05.

## Results

After analysis of available datasets, 40 patient cases could be retrospectively evaluated. Mean age of patients treated with RAIs was 55.9 ± 14.0 years and 47.3 ± 18.5 years for FOE. The gender ratio for RAIs was 70% females and 30% males, for FOE 45% females and 55% males. Evaluation of RAI-supported restorations was based on data raised 18.4 ± 5.7 months after intervention. For restorations of natural roots after FOE, the observation period was calculated to be 43.9 ± 16.4 months. Survival rates for all investigated restorations was 100%. Ceramic chipping was documented for one restoration each (FOE and RAI) within the follow-up period. Additionally orthodontic relapse occurred in three teeth (15%). Detailed information regarding FIPS including means and standard deviations (SD) for both investigators are shown in Table 2. Krippendorff’s alpha coefficients did not reveal unacceptable inter-rater reliabilities regarding the investigators and applicability of FIPS for both concepts, FOE and RAIs (Tables 3 and 4). Due to “occlusion” ratings of FIPS = 2 for all investigated restorations, no statistical evaluation could be carried out in this regard. However, statistical analysis revealed significant differences comparing restorations of natural roots after FOE or supported by RAIs. For both authors (M.W.H.B. and M.B.) significant differences were documented when comparing the concepts of FOE and RAIs regarding “bone” in favor of FOE (*p* < 0.01, Table 2). For M.B. significant differences were also documented regarding “interproximal” (*p* < 0.05) and “mucosa” in favor of FOE (*p* < 0.02, Table 2).

**Table 2:** Summarized mean FIPS scores including standard deviations (SD) for each variable [55]

M.B. / M.W.H.B				
	FOE		RAIs	
*n* = 20 patients each (FOE and RAIs)	Mean	SD	Mean	SD
Interproximal Contacts and papillae	1.7* / 1.6	0.57 / 0.60	1.45* / 1.45	0.51 / 0.51
Occlusion Static and dynamic	2.0 / 2.0	1.0 / 0.0	2.0 / 2.0	0.0 / 0.0
Design Contour and color	1.55 / 1.40	0.61 / 0.68	1.55 / 1.70	0.61 / 0.47
Mucosa Quality and quantity	1.95* / 1.85	0.22 / 0.37	1.65* / 1.60	0.59 / 0.68
Bone X-ray	1.95* / 1.95*	0.22 / 0.22	0.7* / 0.95*	0.73 / 0.83
Total scores	9.2 / 8.8	1.10 / 1.20	7.35 / 7.7	1.27 / 1.49

Abbreviations: (1) M.B. and M.W.H.B.: first and last named authors; (2) RAIs: Root-analogue implants; (3) FOE: Forced orthodontic extrusion

*indicates significant difference between FOE and RAIs (interproximal: *p* < 0.05; mucosa: *p* < 0.02; bone: *p* < 0.01)

**Table 3:**
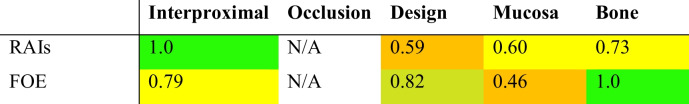
Krippendorff’s alpha coefficients showing inter-rater reliabilities between authors M.B. and M.W.H.B. [58]

Abbreviations: (1) M.B. and M.W.H.B.: first and last named authors; (2) RAIs: Root-analogue implants; (3) FOE: Forced orthodontic extrusion

**Table 4:**
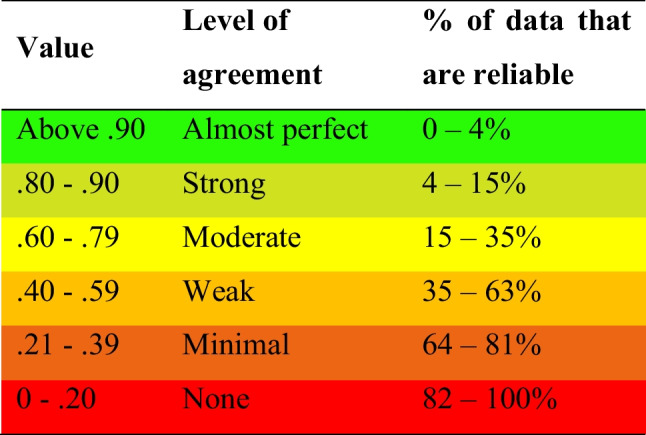
Interpretation guideline following McHugh’s strict classification showing inter-rater reliabilities [58]

## Discussion

To the best knowledge of the authors, the present study is the first that compares restorations supported by RAIs or natural roots preserved by means of FOE. A retrospective data evaluation was performed because both concepts required for comparable basic prerequisites: deeply destroyed teeth that would have been extracted due to their extension of decay in many cases [28, 47]. In addition, little scientific data is available for both treatment options, mainly consisting of case reports and case series [26, 59–69]. Therefore, more clinical data is desirable and has already been demanded [28, 47, 70]. Working hypothesis must be rejected in particular regarding marginal bone levels in favor of the concept of FOE.

FIPS was chosen for evaluations as it combines functional, esthetic and radiographic parameters, while being a simple, self-explaining, reliable, reproducible and quickly applicable score [55, 56]. Although initially developed for comparison of implant-retained restorations it allows the assessment of clinical and functional parameters for both treatment concepts. Moreover, it might document risk factors and might allow for long-term prognosis. In comparison, this is not the case with other assessment measures such as the pink and white esthetic score [71, 72] or the United States Public Health Service (USPHS) criteria [73]. FIPS is therefore a simple and reproducible score for (implant-supported) restorations [55, 56]. Thereby, it should be mentioned, that FIPS was originally developed for implant-retained restorations. However, four out of five parameters can be applied analogously (Table 1). Documented mean scores of 9.2/8.8 ± 1.1/1.2 (FOE) and 7.4/7.7 ± 1.3/1.5 (RAIs, Table 2) represent highly satisfying results regarding investigated cases, especially for restorations of natural roots after FOE. An adapted assessment of bone loss after FOE as described in materials and methods was applicable (Table 1).

Taking a separate look at the sub-parameters of FIPS, for both investigators, evaluated bone loss was significantly higher after immediate placement of RAIs compared to the concept of FOE (*p* < 0.01, Table 2). This result is also supported by the documented “moderate” (RAIs) to “almost perfect” (FOE) inter-rater reliabilities (Tables 3 and 4). For the author M.B. significantly better scores were also achieved regarding “interproximal” (*p* < 0.05) and “mucosa” (*p* < 0.02, Table 2) after utilizing the concept of FOE. These results are supported by “moderate” (FOE) to “almost perfect” (RAIs) inter-rater reliabilities (Tables 3 and 4) regarding "interproximal", though no statistically significant differences were documented for the author M.W.H.B. However, for “mucosa” only weak inter-rater reliabilities were documented (Tables 3 and 4), which should put the interpretation into perspective and may indicate subjective bias.

According to these results, it can be assumed that the concept of FOE seems to prevent marginal bone loss compared to immediate implant installation of RAIs. This tendency in favor of the concept of FOE can also be observed regarding soft tissues, which, however, seems to have a more subjective component than in the assessment of bone. In comparison a mean pink esthetic score of 7.45 ± 1.50, representing highly satisfying results as well, was documented in an extensive follow-up study of milled RAIs in 2020 [28]. These findings are supported by a scoping review, highlighting that RAIs might prevent a loss of alveolar bone volume with maintenance of peri-implant soft tissues leading to an improved esthetic and functional prosthetic result [36]. However, the review was focused on RAIs manufactured from zirconia and a prospective one-year clinical follow-up study documented higher survival rates for milled titanium RAIs compared to milled zirconia RAIs and RAIs manufactured by direct laser metal sintering (DLMS) [31]. This brief illustration of different materials and manufacturing processes highlights the need for further clinical studies on RAIs, especially regarding manufacturing processes and material selection.

Both treatment options are strongly limited by their inclusion criteria as described in the material and methods section. Functional aspects and available occlusal space are particularly important. Regarding RAIs, preservation of surrounding bones during surgery is mandatory. Additionally, its fit can only be checked intraoperatively, after the root has already been removed. Thus, complications can lead to short-term discontinuation of treatment. For the concept of FOE main limitations are patient’s compliance as they are expected to change the orthodontic elastics and losses of the applied fiber-reinforced posts on root surfaces or neighboring teeth. However, no severe complications can be induced, but quite the opposite: FOE can be an alternative in case of absolute contraindications regarding implant therapy [74, 75], limitation of treatment costs [76] and for growing, young patients [77, 78].

Despite possible limitations and complications, it should be noted that conventional restorations with FDPs, RBFDPs or conventional screw-shaped implants are still possible even if RAI-supported restorations or restorations of natural roots after FOE fail. However, regarding the results of marginal bone loss, possible compromised bone volume after RAI loss should be critically kept in mind. No data in this context is available in the literature.

Though bone loss based on two-dimensional x-rays was applied in numerous publications [79, 80], findings should be interpreted with care. Additionally, the retrospective design and no use of standardized radiographs with customized x-ray holders are limiting the meaningfulness of the results. Furthermore, it must be mentioned, that the mean clinical service differed between 18.4 ± 5.7 months (RAIs) and 43.9 ± 16.4 months (FOE). Marginal bone loss in the RAI group might even be higher as reported after the mean service time of restorations utilizing the concept of FOE. Presumably clinical, radiological and esthetic outcomes of restorations after FOE recorded after approx. 1.5 years wouldn’t effect FIPS values negatively compared to after approx. 3.5 years as specified. To minimize subjective bias, all patient cases were assessed by two practitioners independently. Additionally, inter-rater reliabilities were calculated with Krippendorff’s alpha and McHugh’s strict interpretation model was applied [58]. Compared to other interpretations, inter-rater reliability of 0.40 – 0.59 is thereby already described as “weak”, whereas it is described as “fair”, “good” or “moderate” in other interpretation scales. However, it demonstrated, that the “own" procedure tends to be rated as better than the "other" one, respectively. Thus, these results confirm, but also put into perspective, the objectivity of FIPS. This may also highlight the influence of subjective bias especially with regard to studies with a single examiner/practitioner. In this regard, it should be noted in conclusion that all RAI-treatments were performed by the author D.H. and respective follow-up examinations by the author M.W.H.B. Furthermore, all FOE-treatments and follow-up examinations were performed by the author M.B. It would have been more desirable if the assessment by means of FIPS had been carried out by at least a single or multiple completely independent practitioners. However, regarding the additional effort and the specialty of treatment procedures, this was not implemented. In conclusion, this should be kept in mind as source of bias despite calculations and discussion of inter-rater reliabilities.

## Conclusions

It can be concluded that both concepts are equal opportunities in restoring deeply destroyed, i.e. “unrestorable”, teeth showing clinically acceptable results. Nevertheless, they primarily play a role alongside conventional prosthetic treatment options as FDP, rbFDP or an implant-retained single crown. However, especially bone- and tissue-preserving characteristics regarding the concept of FOE are promising. It might also be applicable regarding scheduled socket preservation and subsequent conventional dental implant placement in an adapted workflow [81]. Further long-term data on success, survival, functional and esthetic outcomes are still desirable for both concepts.
